# Redução do Seio Coronário para Tratamento de Angina Refratária: O que Aprendemos 70 Anos após a Cirurgia de Beck?

**DOI:** 10.36660/abc.20250139

**Published:** 2025-12-11

**Authors:** Sara Del Vecchio Ziotti, Luciana Oliveira Cascaes Dourado, Ranil de Silva, Rasha Al-Lamee, Timothy D. Henry, Luiz Antonio Machado Cesar, Carlos Vicente Serrano, Alexandre Antonio Cunha Abizaid, Luis Henrique Wolff Gowdak

**Affiliations:** 1 Hospital das Clínicas da Faculdade de Medicina da Universidade de São Paulo Instituto do Coração São Paulo SP Brasil Instituto do Coração do Hospital das Clínicas da Faculdade de Medicina da Universidade de São Paulo, São Paulo, SP – Brasil; 2 National Heart and Lung Institute Imperial College London London Inglaterra National Heart and Lung Institute, Imperial College London, London – Inglaterra; 3 Royal Brompton and Harefield Hospitals Guy's and St Thomas’ NHS Foundation Trust London Inglaterra Royal Brompton and Harefield Hospitals, Guy's and St Thomas’ NHS Foundation Trust, London – Inglaterra; 4 Imperial College Healthcare National Health Service (NHS) Trust London Inglaterra Imperial College Healthcare National Health Service (NHS) Trust, London – Inglaterra; 5 The Carl and Edyth Lindner Center for Research and Education The Christ Hospital Cincinnati Ohio EUA The Carl and Edyth Lindner Center for Research and Education, The Christ Hospital, Cincinnati, Ohio – EUA

**Keywords:** Angina Pectoris, Circulação Sanguínea, Isquemia Miocárdica

## Abstract

Com o aumento da prevalência das síndromes coronarianas crônicas, muitos pacientes com aterosclerose extensa apresentam angina não controlada, mesmo recebendo a terapia médica otimizada. Isso se aplica especialmente a pacientes que não são candidatos adequados à revascularização cirúrgica ou percutânea. Diversos tratamentos têm sido investigados para o manejo da angina pectoris e, nesse contexto, o redutor do seio coronário surgiu como uma opção terapêutica promissora. Desde a década de 1950, com o início da cirurgia de Beck, o seio venoso coronário tem sido um ponto focal de pesquisa em terapias anti-isquêmicas.

Avanços científicos significativos foram alcançados no estreitamento do seio venoso nas últimas duas décadas. Graças às melhorias tecnológicas em procedimentos minimamente invasivos e aos métodos mais precisos de avaliação do fluxo sanguíneo miocárdico, uma nova opção terapêutica tornou-se disponível para pacientes que sofrem de angina refratária e, possivelmente, de disfunção microvascular.

Nesta revisão, nosso objetivo é examinar os principais conceitos relacionados à angina pectoris e à isquemia miocárdica, destacando o histórico, o racional fisiopatológico e os aspectos técnicos da redução do seio coronário como terapia para angina refratária. Além disso, exploraremos as evidências científicas das últimas décadas, bem como identificaremos lacunas existentes e delinearemos direções futuras de pesquisa sobre esse tratamento emergente.

**Figure:**
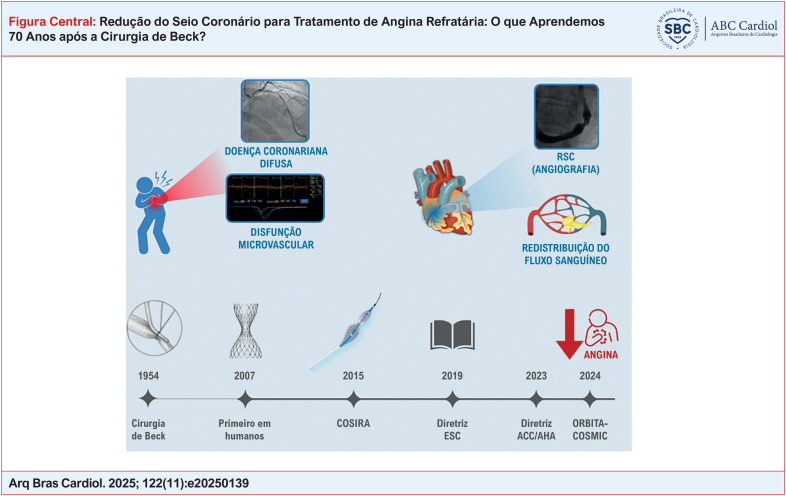
A evolução do redutor do seio coronário e seus marcos históricos para o tratamento de angina refratária. RSC: redutor do seio coronário; ESC: European Society of Cardiology; ACC: American College of Cardiology; AHA: American Heart Association.

Fonte: Autor.

## Introdução

A angina refratária (AR) é caracterizada por dor torácica persistente devido à isquemia miocárdica, apesar da terapia médica otimizada (TMO), que inclui pelo menos duas ou três classes de medicamentos antianginosos.^1-3^ Essa condição geralmente afeta pacientes que não são candidatos adequados para revascularização cirúrgica ou percutânea. A AR pode se manifestar em diversas condições, incluindo doença arterial coronariana (DAC) obstrutiva, angina com artérias coronárias sem lesões obstrutivas (ANOCA), isquemia com artérias coronárias sem lesões obstrutivas (INOCA), cardiomiopatia hipertrófica e insuficiência cardíaca com fração de ejeção preservada.^4^

Embora a prevalência dessa condição não esteja bem estabelecida no Brasil, uma pesquisa populacional com 60.202 indivíduos representando a população brasileira com 18 anos ou mais encontrou uma prevalência de angina moderada/grave de 4,2%.^5^ Na Europa, estima-se que haja entre 30 000 e 50 000 novos casos por ano, enquanto nos Estados Unidos, a prevalência varia de 600 000 a 1,8 milhão de pacientes com AR.^1,6^

A angina crônica é uma condição altamente incapacitante que reduz significativamente a qualidade de vida e está associada a taxas elevadas de transtornos mentais, como a depressão.^7,8^ Ela também leva a taxas mais altas de hospitalização devido aos sintomas isquêmicos, o que impõe um ônus adicional aos sistemas de saúde. A taxa de mortalidade de pacientes com AR é comparável àquela de indivíduos com síndromes coronarianas crônicas não refratárias, estimada em cerca de 4% ao ano.^9,10^ Portanto, os objetivos primários das terapias são aliviar os sintomas isquêmicos, melhorar a tolerância ao exercício e aumentar a qualidade de vida geral.^1,4^

**Figura 1:**
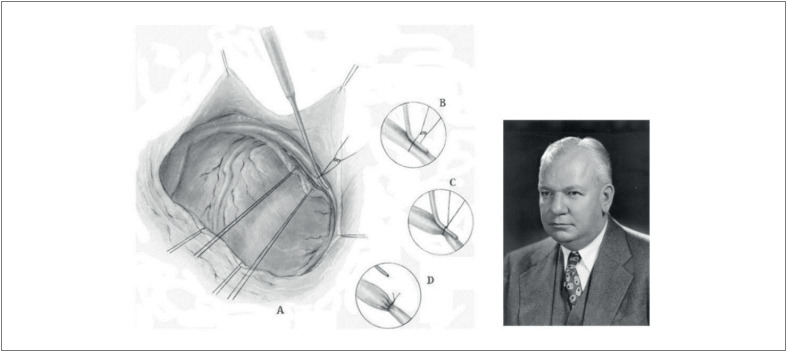
À esquerda, uma ilustração da operação Beck I retirada de seu capítulo na Encyclopedia of Thoracic Surgery, Volume II, Parte Especial 1.9A, mostrando a criação da oclusão parcial do seio coronário. À direita, o Professor Claude S. Beck (1894–1971).

Desde o início do século XX, pesquisadores têm buscado tratamentos eficazes para angina, especialmente desde que as revascularizações miocárdicas experimentais começaram na década de 1930. Em 1954, Claude Beck^11^ publicou um estudo experimental envolvendo cães, no qual avaliou a extensão das áreas isquêmicas, taxas de mortalidade e o fluxo coronariano retrógrado após a oclusão aguda de um ramo principal da coronária esquerda (artéria descendente anterior ou artéria circunflexa).^12^ Isso foi realizado em animais com e sem estreitamento cirúrgico do seio venoso coronário (Figura 1). O autor encontrou uma taxa de mortalidade pós-infarto de 70% no grupo controle, comparada a apenas 27% no grupo de intervenção. Além disso, no grupo de intervenção, a extensão da área infartada foi reduzida em 60% a 70%, e o fluxo coronariano retrógrado foi duplicado nos animais com o seio venoso estreitado em comparação ao grupo controle.

Adaptado de Theruvath et al.^11^

Naquela época, a técnica aberta para reduzir o diâmetro do seio coronário em 60% a 70%, resultando em um lúmen de 3 mm, também foi testada em humanos com DAC obstrutiva em um estudo observacional prospectivo.^13^ O estudo incluiu 137 pacientes acompanhados por um período que variou de seis meses a cinco anos. A taxa de mortalidade foi de 13% no grupo de intervenção, enquanto no grupo controle, que recusou a cirurgia, foi de 30%. Entre os sobreviventes, 90% relataram uma melhora não cega nos sintomas de angina, e houve um aumento de duas vezes na disposição para retornar ao trabalho no período pós-operatório em comparação ao período anterior à cirurgia. No entanto, esses benefícios foram observados antes do advento das técnicas de revascularização cirúrgica e percutânea e da utilização de medicamentos que podem aliviar os sintomas isquêmicos e melhorar o prognóstico a longo prazo.

Mais recentemente, estratégias terapêuticas investigacionais, incluindo novos medicamentos antianginosos,^14,15^ revascularização miocárdica com laser transmiocárdico,^16,17^ terapia celular^18-21^ e revascularização miocárdica com ondas de choque externas,^22,23^ foram testadas com diferentes graus de sucesso em pacientes com AR. No entanto, pacientes com AR frequentemente ficam sem opções de tratamento adequadas,^24^ o que destaca a necessidade urgente de terapias eficazes e motiva um renovado interesse na técnica de estreitamento do seio venoso coronário como método potencial para melhorar a isquemia miocárdica e aliviar os sintomas isquêmicos.

### Redutor do seio coronário

**Figura 2:**
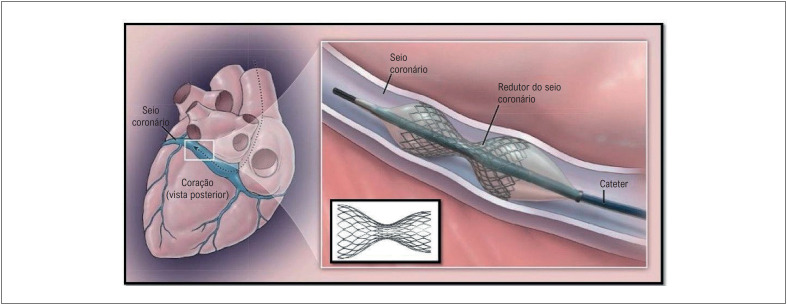
Características e posição anatômica do redutor do seio venoso coronário no seio coronário. A imagem à esquerda ilustra a posição anatômica no seio venoso coronário onde o dispositivo será implantado. À direita, há uma imagem da malha expandida em forma de ampulheta dentro do seio, com seu cateter-balão.

O redutor do seio coronário (RSC) possui uma estrutura em malha semelhante a um *stent* arterial, montado sobre um balão. Quando inflado, adota uma forma de ampulheta com um estreitamento central, conhecido como "pescoço". A extremidade mais distante do operador tem um diâmetro menor do que a extremidade mais próxima do operador. Esse *design* focado na segurança garante que o dispositivo se encaixe bem no seio coronário e minimize o risco de embolização do dispositivo. A Figura 2 mostra o dispositivo e sua posição dentro do coração.^25^

Adaptado de Verheye et al.

### Racional fisiopatológico

Em um coração saudável e não isquêmico, o fluxo sanguíneo é normalmente maior no miocárdio subendocárdico do que no miocárdio subepicárdico. Durante períodos de alta demanda, o sistema nervoso simpático estimula a vasoconstrição das artérias coronárias subepicárdicas, o que melhora a perfusão sanguínea no leito coronário subendocárdico e aumenta a contratilidade muscular. No entanto, quando há obstruções significativas nas artérias coronárias epicárdicas, o fluxo sanguíneo é redistribuído do subendocárdio — onde há maior resistência — para o subepicárdio — que apresenta menor resistência.^26^ Como resultado, a perfusão da região subendocárdica é comprometida, levando à isquemia. Isso pode causar sintomas como angina, redução da contratilidade e disfunção ventricular, aumentando subsequentemente a pressão diastólica final do ventrículo esquerdo (PDFVE).^27^ A PDFVE elevada exerce pressão externa sobre os capilares e arteríolas subendocárdicos, aumentando ainda mais sua resistência vascular, o que agrava o déficit de perfusão e intensifica a isquemia.^27,28^

**Figura 3:**
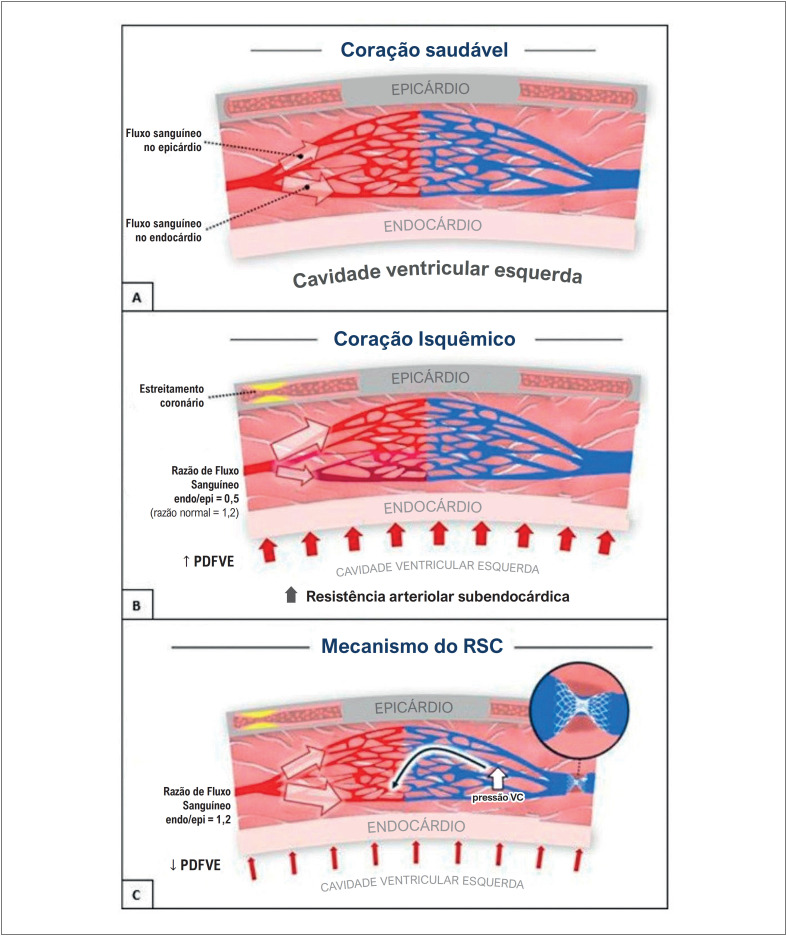
Fisiopatologia da isquemia e do déficit contrátil antes e depois do estreitamento do seio venoso coronário. A) Em um coração não isquêmico, o fluxo sanguíneo no subendocárdio é maior do que no subepicárdio. Durante o exercício, a vasoconstrição seletiva mediada pelo sistema simpático dos vasos subepicárdicos provoca aumento da perfusão subendocárdica e da contratilidade ventricular. B) Em um coração isquêmico devido a uma estenose significativa da artéria coronária epicárdica, a perfusão miocárdica é redistribuída para as camadas subepicárdicas, reduzindo a perfusão do subendocárdio durante o estresse. Essa perfusão reduzida resulta em isquemia, déficit contrátil e elevação da Pressão Diastólica Final do Ventrículo Esquerdo (PDFVE). C) O estreitamento do seio venoso coronário leva ao aumento da pressão retrógrada no sistema venoso coronário, o que dilata as arteríolas, reduz a resistência vascular no subendocárdio e melhora o fluxo sanguíneo. RSC: redutor do seio coronário; VC: venosa capilar.

Quando o seio venoso coronário é estreitado, ocorre um aumento retrógrado da pressão dentro do sistema venoso e capilar. Isso resulta em uma leve vasodilatação arterial, um fator-chave na redução da resistência vascular subendocárdica. Consequentemente, há melhora da isquemia, da função contrátil e dos sintomas associados.^28-31^ Estudos recentes indicam que o RSC também tem impacto direto na microcirculação coronária. Em pacientes com angina e artérias coronárias sem obstruções, a implantação do RSC demonstrou aumentar a reserva de fluxo coronário e reduzir o índice de resistência microvascular após seis meses.^32^ Diversos mecanismos podem contribuir para as alterações na perfusão miocárdica e na resistência microvascular após a implantação do RSC. Esses mecanismos incluem o recrutamento da circulação colateral, o aumento do recrutamento e do diâmetro capilar, a melhora no acoplamento coronário-cardíaco, e neovascularização. Pesquisas atuais estão explorando ativamente esses mecanismos.^33^ A Figura 3 ilustra os mecanismos de perfusão subendocárdica e isquemia antes e depois do estreitamento do seio venoso.

Adaptado de Gindi et al.^28^

### Aspectos técnicos

O procedimento é minimamente invasivo e realizado dentro do sistema venoso. Ele começa com uma punção venosa guiada por ultrassom e colocação de um introdutor, geralmente pela veia jugular interna direita. Um cateter é então avançado sobre um fio-guia até o seio coronário venoso. A pressão média do átrio direito é medida e, se for inferior a 15 mmHg, realiza-se uma venografia do seio coronário para confirmar a anatomia adequada antes da implantação do dispositivo.

**Figura 4:**
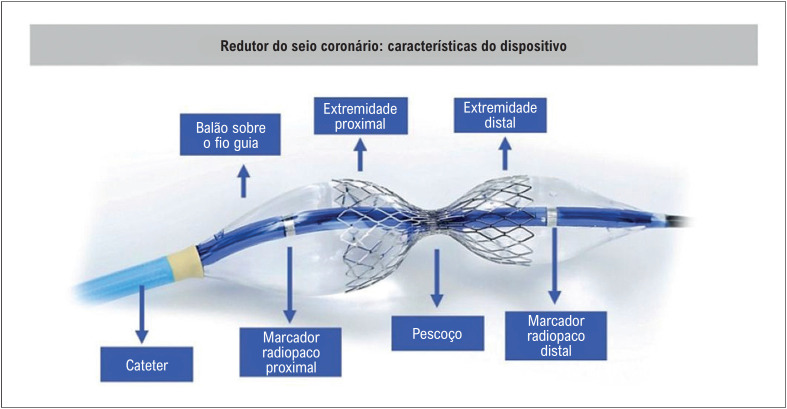
Características do dispositivo de redução do seio coronário. A figura ilustra o cateter-guia com seu balão e a malha expandida em formato de ampulheta, além dos marcadores anatômicos radiopacos.

O dispositivo atualmente aprovado comercialmente com o selo CE na União Europeia e no Reino Unido é o Neovasc Reducer (Neovasc Inc.). Este dispositivo contém três marcadores anatômicos radiopacos: um proximal e um distal ao balão, indicando seus limites, e outro marcador mais proximal, localizado logo fora do balão. O marcador distal ajuda o cardiologista intervencionista a avaliar a posição distal do balão, conforme ilustrado na Figura 4.

Adaptado de Dimitriadis et al.^31^

O dispositivo é montado sobre um balão e está disponível em um único tamanho com capacidade de expansão variável. Ele é inflado de 10% a 20% acima do diâmetro venoso de referência, exigindo aproximadamente de 4 a 6 ATM de pressão. Essa expansão faz com que as extremidades do balão se fixem firmemente no endotélio venoso, com as extremidades distal e proximal expandindo-se para diâmetros de até 9 mm e 14 mm, respectivamente. A área estenótica central possui um diâmetro de aproximadamente 3 mm, representando cerca de 60% a 70% do seu tamanho original (7 a 13 mm). Notavelmente, o dispositivo deve ser idealmente posicionado de 2 a 4 cm distal ao óstio do seio coronário.

**Figura 5:**
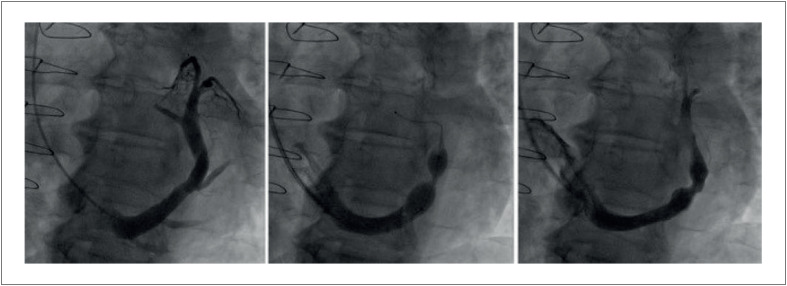
Imagem angiográfica do seio venoso coronário com o dispositivo redutor. A imagem à esquerda ilustra o seio venoso coronário antes da implantação do redutor do seio coronário. As demais imagens mostram a aparência final em formato de ampulheta do redutor após sua implantação.

Após a implantação, inicia-se uma reação inflamatória local, levando à endotelização da malha metálica em suas extremidades, onde há contato com o endotélio. Esse processo geralmente ocorre entre quatro e seis semanas após o procedimento e resulta em um gradiente de pressão, levando à melhora dos sintomas isquêmicos,^4,31,34^ (Figura 5), que apresenta os resultados angiográficos antes e depois da implantação do RSC.

Retirado de Konigstein et al.^37^

Para minimizar o risco de eventos trombóticos, os pacientes são tratados com terapia antiplaquetária dupla (TAPD), utilizando ácido acetilsalicílico (100 mg) e clopidogrel (75 mg) por seis meses após o procedimento. No entanto, é importante observar que ainda não existem estudos que tenham comparado os resultados do RSC com e sem TAPD.^4^

As complicações potenciais do procedimento assemelham-se àquelas associadas ao acesso venoso central, incluindo sangramento, punção arterial, hematoma cervical e pneumotórax, que podem ser manejados de forma conservadora, com transfusão sanguínea ou drenagem torácica, respectivamente. As complicações relacionadas ao dispositivo são incomuns, mas podem incluir dissecção do seio coronário, perfuração, migração do dispositivo e oclusão completa.^35^ Esses eventos podem ser prontamente identificados por fluoroscopia ou avaliados mais detalhadamente por angiotomografia computadorizada. A dissecção ou perfuração do seio coronário geralmente pode ser tratada com medidas conservadoras ou procedimentos minimamente invasivos, como a pericardiocentese. A migração do dispositivo pode ser assintomática ou apresentar sintomas como dor torácica e dispneia, sendo normalmente manejada de forma conservadora, por meio de retirada percutânea ou – caso essas abordagens falhem – por remoção cirúrgica. O risco de migração do dispositivo aumenta quando o diâmetro do seio excede 13 mm ou quando a expansão do balão é insuficiente para ancorar o dispositivo ao endotélio. Embora não tenham sido relatados casos de trombose do dispositivo, há consenso entre especialistas quanto ao uso de TAPD.^4,31,35^

O sistema A-Flux™, um dispositivo inovador desenvolvido pela VahatiCor, está atualmente em avaliação clínica e ainda não está disponível comercialmente. O A-Flux incorpora diversos avanços técnicos em relação aos sistemas expansíveis por balão, que podem melhorar tanto a segurança quanto a eficácia. Ele é construído com uma malha densa de nitinol e está disponível em quatro tamanhos diferentes, permitindo a implantação no segmento específico do seio coronário onde se espera o maior benefício terapêutico. A estrutura densa de nitinol foi projetada para promover uma modulação rápida do fluxo por meio de uma resposta acelerada de endotelização. Além disso, o sistema de entrega permite uma força de implantação crônica e externa reduzida, fixando o dispositivo no local sem risco de dissecção ou ruptura do seio coronário induzida por balão.^36^

Até o momento, nem o A-Flux™ nem o Neovasc Reducer™ está disponível no Brasil. O Neovasc Reducer™ está atualmente passando por um ensaio clínico aprovado pela FDA nos Estados Unidos.

### Evidência científica

Numerosos estudos demonstraram a segurança e a eficácia de várias intervenções na redução da intensidade e frequência da angina, tanto a curto quanto a longo prazo. Essa evidência é derivada de uma combinação de estudos não randomizados e não cegos, bem como de estudos randomizados e cegos.

Inicialmente, Banai et al.^34^ revisitaram o conceito de Claude Beck e realizaram estudos pré-clínicos em porcos com estenose da artéria circunflexa proximal.^34,37^ Esses estudos avaliaram condições com e sem isquemia utilizando ecocardiografia sob estresse. Os resultados demonstraram uma redução na extensão e na gravidade da área isquêmica, conforme avaliado por ecocardiografia com dobutamina. Além disso, os estudos confirmaram a viabilidade e a segurança do procedimento. A análise histológica realizada nos animais após um a seis meses mostrou uma resposta inflamatória local aceitável, completa endotelização do dispositivo e neointima estável.

O mesmo grupo de pesquisa publicou o primeiro estudo clínico sobre a redução do seio coronário por cateterismo em humanos, envolvendo 15 pacientes.^34^ Trata-se de um ensaio clínico aberto e não randomizado, com o objetivo de avaliar a segurança e a viabilidade da implantação percutânea do RSC em pacientes com AR, classificados como classe funcional 2 ou superior pela *Canadian Cardiovascular Society* (CCS). O estudo não relatou eventos adversos significativos durante o período de acompanhamento de seis meses. A eficácia foi medida pela classe CCS, enquanto a isquemia foi avaliada por meio de ecocardiografia sob estresse com dobutamina e tomografia por emissão de pósitrons (PET) com tálio. A média da classe de angina diminuiu significativamente de 3,07 para 1,64 (p < 0,001), e foram observadas melhorias tanto na extensão quanto na gravidade da isquemia (p = 0,004 e 0,042, respectivamente).

Konigstein et al.^38^ conduziram um estudo não randomizado envolvendo 23 pacientes e demonstraram melhorias na angina, isquemia e contratilidade ventricular esquerda em pacientes com AR.^38^ A intervenção foi tecnicamente bem-sucedida em 21 dos 23 pacientes. O benefício clínico estatisticamente significativo foi documentado por meio da redução na média da pontuação CCS de 3,3 na linha de base para 2,0 aos seis meses, aumento na duração do exercício de 3:16 para 5:16 minutos, e redução nas pontuações somadas de estresse e diferença na cintilografia miocárdica com tálio-201 (21,5±10 vs. 13,2±9 e 11,1±6 vs. 4,7±4, respectivamente), demonstrando a importância da técnica como uma nova estratégia terapêutica para o tratamento de pacientes com AR.

O estudo COSIRA,^25^ publicado em 2015, foi o primeiro estudo randomizado, controlado por placebo, que incluiu 104 indivíduos com AR classificados como classe 3 e 4 da CCS, apresentando isquemia miocárdica.^25^ O estudo constatou que o dispositivo levou à melhora de pelo menos uma classe funcional de angina em 71% dos pacientes do grupo de intervenção (37 de 52), em comparação com 42% no grupo placebo (22 de 52) (p = 0,003). Além disso, pelo menos duas classes de angina melhoraram em 35% do grupo de intervenção (18 de 52) versus 15% no grupo placebo (8 de 52) (p = 0,02). Embora seja importante observar a taxa significativa de melhora no grupo placebo, a intervenção mostrou uma redução 2,4 vezes maior na classe de angina em comparação ao controle, indicando sua superioridade como tratamento antianginoso. Uma análise post-hoc da coorte do estudo COSIRA também revelou melhorias na qualidade de vida e na tolerância ao exercício dentro do grupo de intervenção, reforçando ainda mais a eficácia da intervenção.^39^

Em outro estudo observacional unicêntrico único, Giannini et al.^40^ avaliaram 50 pacientes com AR e isquemia documentada, com foco na angina, tolerância ao exercício e qualidade de vida aos quatro e 12 meses de acompanhamento.^40^ O procedimento alcançou uma taxa de sucesso técnico de 100%, sem relatos de eventos adversos relacionados ao dispositivo. Entre os pacientes, 80% apresentaram redução de pelo menos uma classe CCS, e 40% apresentaram redução de pelo menos duas classes, com uma redução média da classe para 1,67 ± 0,83 vs. 2,98 ± 0,52 (p < 0,001) no acompanhamento de quatro meses em comparação ao basal. Também foram observadas melhorias significativas na qualidade de vida e na tolerância ao exercício, conforme medido pelo teste de caminhada de seis minutos, além da redução na necessidade de medicamentos antianginosos. Importante destacar que essas melhorias foram sustentadas ao longo do tempo, mesmo após o primeiro ano de acompanhamento.

O mesmo grupo investigou mais profundamente os efeitos sintomáticos do RSC em pacientes com angina devido à ANOCA, por meio de um estudo observacional prospectivo envolvendo oito pacientes classificados como classes 3 e 4 de angina segundo a CCS.^41^ O procedimento foi bem-sucedido em todos os casos, sem relatos de eventos adversos. A classe CCS melhorou de uma mediana de 3,0 para 1,5 (P = 0,014). Após um ano de acompanhamento, três dos cinco pacientes avaliados continuaram a apresentar benefício clínico. Além disso, foram observadas melhorias no teste de caminhada de seis minutos e nos sintomas de angina, conforme medido pelo Questionário de Angina de Seattle (SAQ).^41^

Em um estudo de registro prospectivo envolvendo 48 pacientes com AR classes 3 e 4 da CCS, Konigstein et al.^42^ avaliaram melhorias na angina utilizando o SAQ, a classe funcional da CCS e avaliações objetivas de angina e isquemia. Essas avaliações incluíram testes de esforço em esteira, ecocardiografia com dobutamina e testes de caminhada de seis minutos. Os resultados demonstraram que 85% dos pacientes (33 de 39) apresentaram melhora de pelo menos uma classe funcional da CCS, enquanto 48% (19 de 39) tiveram melhora de pelo menos duas classes. Em relação à função ventricular, a fração de ejeção do ventrículo esquerdo (FEVE) sob estresse aumentou de 51±10 para 56±10 (p = 0,004), e o índice de movimento parietal melhorou de 1,58±0,40 para 1,37±0,30 (p = 0,004), indicando efeitos positivos na função ventricular. Nenhum evento relacionado ao procedimento ou ao dispositivo foi relatado durante um período médio de acompanhamento de 12,5 meses.

Posteriormente, Tzanis et al.^43^ conduziram um estudo focado em sintomas e função ventricular em 18 pacientes, encontrando uma redução de uma classe na CCS em 84% dos indivíduos após quatro meses de acompanhamento.^43^ Também foram observadas melhorias significativas na função ventricular esquerda, com aumentos na FEVE e reduções nos volumes sistólico e diastólico finais do ventrículo esquerdo. Notavelmente, metade dos pacientes apresentava FEVE reduzida na linha de base, mostrando um aumento ainda maior na FEVE em comparação com aqueles com FEVE preservada (aumento de 11,3 para FEVE reduzida versus 3,8 para FEVE preservada; p = 0,029).

Outro estudo sobre a gravidade da obstrução coronariana foi realizada por Zivelonghi et al.^44^ em um estudo multicêntrico retrospectivo envolvendo 205 pacientes, dos quais 103 (50,2%) apresentavam oclusão total coronariana (OTC) não revascularizada. Isso permitiu comparar os efeitos do RSC em pacientes com e sem lesões do tipo OTC. Inicialmente, a classe CCS era 3,0±0,5 no grupo OTC e 3,1±0,6 no grupo não-OTC (p = 0,45). No acompanhamento de seis meses, as classes CCS melhoraram para 1,6±0,9 e 2,0±1,1, respectivamente (p < 0,01), com melhora significativamente maior observada no grupo OTC (1,4±0,9 vs. 1,1±1,0; p = 0,01). A melhora da angina foi observada em todos os territórios, incluindo 70% para CTO da artéria coronária direita (35 pacientes), 85% para OTC da artéria descendente anterior esquerda (17 pacientes) e 82% para OTC da artéria circunflexa (27 pacientes). Concluiu-se que pacientes com lesões OTC podem responder melhor ao RSC do que pacientes sem OTC, independentemente do território afetado. Nenhuma complicação relacionada ao procedimento foi relatada, embora tenha ocorrido embolização do dispositivo em dois casos (1%), nos quais o dispositivo foi recuperado com sucesso e o procedimento foi concluído com a implantação de um segundo RSC. Eventos adversos semelhantes foram observados em ambos os grupos do estudo, incluindo um total de sete mortes cardíacas (3,4%) e 30 revascularizações adicionais (14,6%) devido à angina persistente.

Os benefícios em longo prazo do RSC também foram explorados no estudo REDUCER-I, um estudo observacional multicêntrico com braços retrospectivos (do ensaio COSIRA) e prospectivos.^45^ Esse foi um estudo multicêntrico observacional que incluiu 228 pacientes com AR nas classes CCS 2 a 4 – 180 no braço prospectivo e 48 no braço retrospectivo do ensaio COSIRA. O objetivo do estudo foi avaliar a segurança e a eficácia do RSC e seu papel na melhora da gravidade da angina e da qualidade de vida após dois anos de acompanhamento. A taxa de sucesso foi de 99%. A média da classe CCS diminuiu de 2,8±0,6 para 1,8±0,7 após dois anos; pelo menos uma classe CCS melhorou em 82% dos pacientes, e pelo menos duas classes melhoraram em 31% dos pacientes. Também foram observados aumentos significativos no teste de caminhada de seis minutos e na duração do exercício. A taxa de não respondedores foi de 24% após um ano e 17% após dois anos. Melhoras foram observadas em todos os domínios avaliados seis e doze meses após o tratamento. O estudo destacou a melhora sustentada na limitação da angina do sexto mês até dois anos de acompanhamento.

O estudo RESOURCE representa o maior registro multicêntrico, avaliando procedimentos de implantação do RSC ao longo de 10 anos (de 2010 a 2020).^35^ Ele incluiu dados de 658 pacientes com AR, dos quais 89,4% estavam nas classes 3 ou 4 da CCS. O acompanhamento médio desses pacientes foi de 502 dias. O estudo avaliou diversos desfechos, incluindo melhorias na angina, taxa de sucesso dos procedimentos, complicações relacionadas ao procedimento e eventos cardiovasculares maiores (MACE). A intervenção foi bem-sucedida em 96,7% dos casos e suspensa em 3% devido à anatomia inadequada ou complicações. Ainda assim, não houve eventos graves nas primeiras 48 horas. Complicações por procedimento ocorreram em 5,7% dos casos (38 de 663 procedimentos). Entre essas complicações, as mais comuns foram: embolização do dispositivo, hematoma cervical, dissecção do seio coronário, desconexão do dispositivo do cateter-guia antes da implantação e perfuração do seio coronário. Importante destacar que nenhuma dessas complicações resultou em mortes ou infartos relacionados ao procedimento, nem levaram ao aumento da mortalidade ou dos eventos MACE durante o acompanhamento. O desfecho primário de eficácia, que era a redução de pelo menos duas classes da angina segundo a classificação da CCS, foi alcançado em 39,7% dos pacientes, enquanto uma melhora de pelo menos uma classe foi observada em 76,0%. A taxa de não respondedores foi de 24%. A frequência de MACE foi de 14,6%, e as taxas de mortalidade em 1, 3 e 5 anos foram de 4,0%, 13,7% e 23,4%, respectivamente. Esses números são comparáveis às taxas de mortalidade observadas em pacientes com AR na coorte OPTIMIST.^9^

Em uma meta-análise recente de nove estudos (n=846), incluindo um ensaio clínico randomizado controlado por placebo (COSIRA), Hochstadt et al.^46^ demonstraram uma eficácia combinada do RSC de 76% para melhora dos sintomas em pelo menos uma classe da CCS de DAC e 40% para pelo menos duas classes. Além disso, a distância média percorrida durante o teste de caminhada de seis minutos aumentou em 45,5 metros, superando a melhora observada em estudos sobre insuficiência cardíaca (30,1 metros). Quanto à segurança, o procedimento foi bem-sucedido em 98% dos casos. Apenas 3% dos pacientes apresentaram complicações, como perfuração/dissecção do seio coronário, embolização ou deslocamento do dispositivo, e infarto periprocedural. Importante destacar que nenhuma dessas complicações resultou em tamponamento cardíaco ou morte, confirmando ainda mais a alta taxa de sucesso e segurança do procedimento.

Em 2024, os resultados tão aguardados do estudo ORBITA-COSMIC foram publicados.^47^ Este estudo clínico histórico, randomizado, duplo-cego e controlado por placebo envolveu 51 pacientes e teve como objetivo avaliar o impacto do RSC na isquemia miocárdica. A análise incluiu a quantificação do fluxo sanguíneo miocárdico por meio de ressonância magnética cardíaca (RMC) sob estresse com adenosina, testes de esforço físico e o uso de um questionário de sintomas em tempo real pelo aplicativo ORBITA-app. Os desfechos primários incluíram o fluxo sanguíneo miocárdico em segmentos isquêmicos, conforme determinado pela CMR, e o principal desfecho sintomático foi o número diário de episódios de angina. O estudo não demonstrou melhora no fluxo sanguíneo miocárdico para os segmentos isquêmicos seis meses após a implantação do RSC. No entanto, houve uma melhora proporcional no fluxo sanguíneo endocárdico em comparação ao epicárdico sob estresse no grupo RSC, e essa diferença foi observada entre segmentos isquêmicos e não isquêmicos. Os pacientes do grupo RSC relataram menos episódios diários de angina no acompanhamento de seis meses, com início por volta de 70 dias após o procedimento, o que condiz com o tempo necessário para a endotelização do dispositivo e a formação do gradiente de pressão, que começa cerca de seis semanas após a implantação do RSC. Nos desfechos secundários, houve melhora na frequência da angina com base no SAQ, embora não tenham sido encontradas diferenças estatísticas na qualidade de vida e na tolerância ao exercício. Neste estudo, a melhora dos sintomas em pacientes com AR não foi acompanhada por uma melhora geral na perfusão dos segmentos isquêmicos. Por outro lado, houve melhora na perfusão subendocárdica dentro dos segmentos isquêmicos, apoiando a teoria de que o dispositivo redistribui o fluxo sanguíneo em direção ao endocárdio.^48^ Esses achados têm implicações importantes para a compreensão da isquemia miocárdica e o papel potencial do RSC em seu tratamento.

### Seleção dos pacientes

A evidência científica que respalda o RSC indica que ele é adequado para o tratamento da angina pectoris ou equivalentes isquêmicos em pacientes com síndromes coronarianas obstrutivas crônicas. Esse tratamento é especificamente indicado para pacientes com AR que não responderam à TMO, a qual inclui pelo menos duas ou três classes de medicamentos antianginosos, e que não possuem opções de revascularização cirúrgica ou percutânea, ou têm essas opções limitadas.^4^

Apesar dos benefícios observados em diferentes estudos clínicos, de 20 a 30% dos pacientes podem não responder de maneira positiva a esse tratamento.^49^ Diversos fatores podem contribuir para uma resposta clínica desfavorável,^50,51^ incluindo dor torácica não anginosa, angina causada por isquemia no território da artéria coronária direita, dispneia aos esforços decorrente de insuficiência cardíaca e cobertura inadequada da estrutura metálica do dispositivo, o que pode levar à má endotelização dos suportes do dispositivo.

Além disso, uma drenagem venosa miocárdica alternativa bem desenvolvida por meio da rede de Thebesius pode afetar os resultados.^52^ Um marca-passo biventricular para terapia de ressincronização cardíaca pode complicar o procedimento do ponto de vista técnico, já que seus eletrodos são posicionados próximos ao óstio do seio venoso, aumentando o risco de complicações agudas e possível falha na inserção do dispositivo. Ainda, insuficiência cardíaca avançada ou sintomática com pressão elevada no átrio direito (superior a 15 mmHg) pode indicar descompensação cardíaca, reduzindo ainda mais a eficácia da intervenção. A Tabela 1 apresenta os fatores positivos e negativos na seleção do paciente ideal que pode se beneficiar do tratamento com RSC.

**Tabela 1:** Critérios para selecionar os melhores candidatos ao uso do redutor de seio coronário

Candidatos adequados	Candidatos não adequados
Angina estável (há mais de 3 meses), classe CCS ≥ 2 apesar da terapia médica otimizada	Dor torácica não coronariana (doença valvar cardíaca grave, insuficiência cardíaca, dor não cardíaca)
Evidência de isquemia reversível	Ausência de isquemia induzida por estresse
Isquemia miocárdica no território da artéria coronária esquerda	Isquemia miocárdica exclusivamente no território da artéria coronária direita
Fração de ejeção do ventrículo esquerdo > 30%	Fração de ejeção do ventrículo esquerdo < 30% ou insuficiência cardíaca classe NYHA ≥ 3
Pressão média do átrio direito ou pressão venosa central < 15 mmHg	Pressão média do átrio direito ou pressão venosa central > 15 mmHg
Eletrodos de marcapasso implantados no átrio direito há mais de 3 meses	Eletrodos de marcapasso implantados no átrio direito há menos de 3 meses
	Eletrodos de terapia de ressincronização cardíaca no seio coronário
	Síndrome coronariana aguda recente, intervenção coronária percutânea ou cirurgia de revascularização miocárdica há menos de 3 meses

Adaptado de Konigstein et al.^52^ CCS: *Canadian Cardiovascular Society*, NYHA: *New York Heart Association*.

### Perspectivas

Estudos observacionais anteriores sugerem que o RSC pode oferecer benefícios clínicos para pacientes com ANOCA e disfunção microvascular coronariana. Esses benefícios incluem alívio dos sintomas e melhora da função ventricular em pacientes com insuficiência cardíaca com fração de ejeção preservada e reduzida. Isso abre um leque fascinante de novas opções terapêuticas potenciais para pacientes que sofrem de síndromes coronarianas crônicas não obstrutivas e miocardiopatias não coronarianas, como a miocardiopatia hipertrófica.

Vários ensaios clínicos listados na Tabela 2 estão atualmente em andamento, cada um desempenhando um papel crucial no avanço da nossa compreensão e dos benefícios do RSC em diferentes grupos de pacientes com AR.

**Tabela 2:** Estudos envolvendo redutor do seio coronário em andamento

Nome do estudo	País	Tipo de estudo	População	Desfechos primários	NCT
LSSRR – Lower Silesia Sinus Reducer Registry^53^	Polônia	Observacional	Pacientes com angina pectoris crônica incapacitante e refratária (classes CCS 2–4), apesar da terapia médica antianginosa máxima tolerada, que foram submetidos à implantação do RSC	Classe CCS, escore SAQ-7, teste de caminhada de 6 minutos, e ecocardiografia 1 anos após o implante do RSC	NCT06288165
Impact of Coronary Sinus Flow Reducer on Coronary Microcirculation and Myocardial Ischemia^54^	Croácia	Intervencionista, grupo único	Pacientes com doença arterial coronariana e angina pectoris refratária que não são elegíveis para revascularização coronariana.	Avaliação da reserva de fluxo coronariano e índice de resistência microcirculatória antes e depois da implantação do RSC.	NCT06266065
Use of the Neovasc Coronary Sinus Reducer System for the Treatment of Refractory Angina Pectoris in Patients With Angina Class 3-4 Who Are Not Candidates for Revascularization (Reducer)^55^	Israel	Intervencionista, grupo único	Doença arterial coronariana sintomática com angina pectoris crônica refratária classificada como grau III ou IV pela CCS, apesar da tentativa de terapia médica otimizada nos trinta dias anteriores à triagem Não candidato à revascularização cirúrgica ou por ICP Isquemia reversível da parede do ventrículo esquerdo demonstrada por ecocardiografia com estresse por dobutamina ou por cintilografia com tálio (Thallium SPECT)	Redução de duas ou mais classes da CCS em relação ao valor basal até seis meses após o procedimento	NCT01566175
COSIMA: COronary SInus Reducer for the Treatment of Refractory Microvascular Angina (COSIMA)^56^	Alemanha	Randomizado, controlado, grupos paralelos (RSC vs. TMO)	Síndrome coronariana crônica (incluindo pacientes com equivalentes anginosos) com angina refratária classe III-IV pela CCS, apesar da terapia médica guiada por diretrizes Evidência de isquemia reversível em testes não invasivos Evidência de doença microvascular diagnosticada de forma invasiva por pelo menos um dos seguintes critérios: IMR >25 e/ou RFC <2,0 com FFR >0,8	Proporção de pacientes com melhora de ≥ 2 classes de angina pela CCS seis meses após a implantação do RSC	NCT04606459
IMR Evaluation in Patients With Coronary Sinus Reducer Implantation (INROAD Study)^57^	Itália	Intervencionista, grupo único	Angina crônica refratária a terapias médicas e intervencionistas Pelo menos uma artéria coronária aberta (excluindo a artéria coronária direita) onde será realizada a avaliação do IMR	Variação ≥ 20% no valor do IMR quatro meses após a implantação do RSC em comparação ao valor basal	NCT05174572
Efficacy of the COronary SInus Reducer in Patients With Refractory Angina II (COSIRA-II)^58^	Estados Unidos	Intervencionista, randomizado, duplo-cego, controlado por procedimento simulado (sham)	Doença arterial coronariana sintomática com angina pectoris refratária persistente, classificada como Grau III ou IV pela CCS, apesar do tratamento médico otimizado (OMT) na dose máxima tolerada Evidência de isquemia reversível induzida por exercício ou farmacologicamente, avaliada por ecocardiograma de estresse, estudo nuclear, PET, ressonância magnética de perfusão, tomografia de perfusão, RFF derivada da TC, RFF, iRF ou outros testes não hiperêmicos na distribuição da artéria coronária esquerda	Alteração na duração total do exercício em uma avaliação de teste de esforço em esteira com protocolo Bruce modificado no grupo de tratamento em comparação ao grupo controle simulado Taxa de ocorrência de um desfecho composto de morte, IM, derrame pericárdico que requer intervenção cirúrgica ou percutânea, embolização do dispositivo ou sangramento BARC tipo 3 ou 5 no grupo de tratamento em comparação ao grupo controle simulado	NCT05102019
Coronary Sinus Reducer Implantation in Patients With Ischaemia and Non-obstructed Coronary Arteries and Coronary Microvascular Dysfunction (REMEDY-PILOT)^59^	Reino Unido	Intervencionista, randomizado, duplo-cego, controlado por procedimento simulado (sham)	Angina sintomática persistente, Classe II-IV da CCS, por ≥3 meses, apesar do tratamento de base com pelo menos dois medicamentos antianginosos na dose máxima tolerada Artérias coronárias sem obstrução, com estenoses epicárdicas ≤30% demonstradas na angiografia coronária Hipoperfusão subendocárdica circunferencial induzida por estresse na ressonância magnética cardíaca (CMR), com RPM global < 2,5	Alteração aos 6 meses após a randomização, em comparação ao valor basal, na MPR global quantitativa avaliada por ressonância magnética cardíaca	NCT05492110
Myocardial Ischemia After Coronary Sinus Reduction Stent Implantation (MICS-Reduce)^60^	Noruega	Observacional	DAC e angina refratária	Change in myocardial flow reserve on ^15^O-H_2_O PET/CT	NCT06033495

RSC: redutor do seio coronário, CCS: Canadian Cardiovascular Society, NYHA: New York Heart Association, SAQ-7: Questionário de Angina de Seattle – 7 itens; CAD: doença arterial coronariana. ICP: intervenção coronariana percutânea; SPECT: tomografia computadorizada por emissão de fóton único; TMO: terapia médica otimizada; IMR: índice de resistência microvascular; RFC: reserva de fluxo coronariano; RFF: reserva de fluxo fracionada; PET: tomografia por emissão de pósitrons; TC: tomografia computadorizada; iRF: índice de Reserva de Fluxo; RMC: resistência microvascular coronariana; RPM: reserva de perfusão miocárdica.

## Conclusão

O RSC é um dispositivo inserido por via percutânea por meio de um procedimento minimamente invasivo. É seguro e eficaz para controlar sintomas isquêmicos em pacientes com AR que não são candidatos à revascularização cirúrgica ou percutânea e que estão recebendo a melhor terapia médica disponível. O dispositivo, com sua segurança e eficácia comprovadas, acredita-se que funcione ao aumentar retrogradamente a pressão no sistema venoso coronário, o que ajuda a melhorar a perfusão arteriocapilar no miocárdio subendocárdico.

Dados cegos indicam melhora da angina com a implantação do RSC. O dispositivo também melhora a qualidade de vida de pacientes que não têm outras opções terapêuticas. No entanto, é fundamental observar que de 20 a 30% dos pacientes podem não responder ao tratamento. Portanto, o procedimento deve ser cuidadosamente indicado com base em avaliações clínicas e anatômicas detalhadas. Além disso, pequenos estudos não cegos sugerem que o RSC pode melhorar a perfusão e os sintomas de angina em pacientes com ANOCA e disfunção microvascular coronariana, além de potencialmente aumentar a contratilidade miocárdica em casos de insuficiência cardíaca. Entretanto, é essencial a realização de mais estudos randomizados e controlados por placebo para avaliar e validar de forma mais robusta a eficácia do RSC e avançar na compreensão desse tratamento promissor.

Finalmente, após 70 anos desde a primeira redução do seio coronário realizada pela cirurgia de Beck, os cientistas desenvolveram um procedimento minimamente invasivo que foi testado com sucesso em ensaios clínicos randomizados para o tratamento da angina. Esse procedimento foi recentemente incluído nas diretrizes clínicas. A Figura Central representa essa evolução, destacando os principais marcos.

## Data Availability

Os conteúdos estão disponíveis: - doi:10.1016/j.jtcvs2014.06.032 - doi:10.1056/NEJMoa1402556 - doi:10.1016/j.carrev.2022.01.003 - doi:10.1016/j.carrev.2023.07.008 - doi:10.1093/eurhearttj/ehx486
