# Supplementary material for: Redução do Seio Coronário para Tratamento de Angina Refratária: O que Aprendemos 70 Anos após a Cirurgia de Beck?
Source: Arq Bras Cardiol. 2025 Dec 11;122(11):e20250139. [Article in Portuguese] doi: 10.36660/abc.20250139 (PMC12978284; doi:10.36660/abc.20250139)
Supplement: Translation

# Translation: Coronary Sinus Reduction for the Treatment of Refractory Angina: What Have We Learned after 70 Years of the Beck Surgery?

**DOI:** 10.36660/abc.20250139i

**Authors:** Sara Del Vecchio Ziotti, Luciana Oliveira Cascaes Dourado, Ranil de Silva, Rasha Al-Lamee, Timothy D. Henry, Luiz Antonio Machado Cesar, Carlos Vicente Serrano, Alexandre Antonio Cunha Abizaid, Luis Henrique Wolff Gowdak

**Affiliations:** 1 São Paulo SP Brazil Instituto do Coração do Hospital das Clínicas da Faculdade de Medicina da Universidade de São Paulo, São Paulo, SP – Brazil; 2 London United Kingdom National Heart and Lung Institute, Imperial College London, London – United Kingdom; 3 London United Kingdom Royal Brompton and Harefield Hospitals, Guy's and St Thomas’ NHS Foundation Trust, London – United Kingdom; 4 London United Kingdom Imperial College Healthcare National Health Service (NHS) Trust, London – United Kingdom; 5 Cincinnati Ohio EUA The Carl and Edyth Lindner Center for Research and Education, The Christ Hospital, Cincinnati, Ohio – EUA

**Keywords:** Angina Pectoris, Blood Circulation, Myocardial Ischemia

## Abstract

With the increasing prevalence of chronic coronary syndromes, many patients with extensive atherosclerosis experience uncontrolled angina, even while receiving optimal medical therapy. This is especially true for patients who are not suitable candidates for surgical or percutaneous revascularization. Numerous treatments have been investigated for managing angina pectoris, and in this context, the coronary sinus reducer has emerged as a promising therapeutic option. Since the 1950s, beginning with Beck's surgery, the coronary venous sinus has been a focal point of research in anti-ischemic therapies.

Significant scientific advances have been made in narrowing the venous sinus in the past two decades. Thanks to technological improvements in minimally invasive procedures and better methods for assessing myocardial blood flow, a new therapeutic option has become available for patients suffering from refractory angina and, possibly, for microvascular dysfunction.

In this review, we aim to examine key concepts related to angina pectoris and myocardial ischemia, highlighting the historical background, pathophysiological rationale, and technical aspects of coronary sinus reduction as a therapy for refractory angina. Additionally, we will explore the scientific evidence from recent decades, as well as identify existing gaps and outline future research directions concerning this emerging treatment.

**Figure:** The evolution of coronary sinus reduction and key milestones in the treatment of refractory angina. CSR: Coronary Sinus Reducer; ESC: European Society of Cardiology; ACC: American College of Cardiology; AHA: American Heart Association.

Source: Author.

## Introduction

Refractory angina (RA) is characterized by persistent chest pain due to myocardial ischemia despite optimal medical therapy (OMT) that includes at least two or three classes of anti-anginal drugs.^1-3^ This condition typically affects patients who are not suitable candidates for surgical or percutaneous revascularization. RA can manifest across a range of conditions, including obstructive coronary artery disease (CAD), angina with non-obstructive coronary arteries (ANOCA), ischemia with non-obstructive coronary arteries (INOCA), as well as hypertrophic cardiomyopathy and heart failure with preserved ejection fraction.^4^

Although the exact prevalence rates of this condition are not well-established in Brazil, a population survey of 60,202 individuals representing the Brazilian population aged 18 years and older found a prevalence of moderate/severe angina of 4.2%.^5^ In Europe, it is estimated that there are between 30,000 and 50,000 new cases per year, while in the United States, the prevalence ranges from 600,000 to 1.8 million patients with RA.^1,6^

Chronic angina is a highly disabling condition that significantly reduces quality of life and is associated with increased rates of mental health disorders, such as depression.^7,8^ It also leads to higher hospitalization rates due to ischemic symptoms, which places an additional burden on health systems. The mortality rate for patients with RA is comparable to that of individuals with non-refractory chronic coronary syndromes, estimated at around 4% per year.^9,10^ Therefore, the primary goals of therapies are to relieve ischemic symptoms, improve exercise tolerance, and enhance overall quality of life.^1,4^

Since the beginning of the 20^th^ century, researchers have searched for effective treatments for angina, particularly since experimental myocardial revascularizations began in the 1930s. In 1954, Claude Beck^11^ published an experimental study involving dogs, where he evaluated the extent of ischemic areas, mortality rates, and retrograde coronary flow following the acute occlusion of a major left coronary branch (anterior descending or circumflex arteries).^12^ This was performed in animals with and without surgical narrowing of the coronary venous sinus (Figure 1). The author found a post-infarction mortality rate of 70% in the control group, compared to only 27% in the intervention group. Additionally, in the intervention group, the extent of the infarcted area was reduced by 60% to 70%, and retrograde coronary flow was doubled in animals with a narrowed venous sinus compared to the control group.

**Figure 1:** On the left, an illustration of the Beck I operation from his chapter in the Encyclopedia of Thoracic Surgery, Volume II, Special Part 1.9A showing the creation of partial coronary sinus occlusion. On the right, Professor Claude S. Beck (1894-1971).

Adapted from Theruvath et al.^11^

At that time, the open technique for reducing the coronary sinus diameter by 60% to 70%, resulting in a lumen of 3 mm, was also tested in humans with obstructive CAD in a prospective observational study.^13^ The study included 137 patients followed for a period ranging from six months to five years. The mortality rate was 13% in the intervention group, while it was 30% in the control group, which refused surgery. Among the survivors, 90% reported an unblinded improvement in their angina symptoms, and there was a two-fold increase in willingness to return to work in the postoperative period compared to before the surgery. However, these benefits were noted before the advent of surgical and percutaneous revascularization techniques and the utilization of medications that can alleviate ischemic symptoms and improve long-term prognosis.

More recently, investigational therapeutic strategies, including new antianginal drugs,^14,15^ transmyocardial laser revascularization,^16,17^ cell therapy,^18-21^ and external shockwave myocardial revascularization,^22,23^ have been tested with different degrees of success in patients with RA. However, patients with RA are often left with no suitable treatment options,^24^ underscoring the urgent need for effective therapies and prompting a renewed interest in the technique of coronary venous sinus narrowing as a potential method to improve myocardial ischemia and alleviate ischemic symptoms.

### Coronary sinus reducer

The coronary sinus reduces (CSR) has a mesh structure akin to an arterial stent, which is mounted on a balloon. When inflated, it adopts an hourglass shape with a central narrowing, known as the neck. The upstream end (further from the operator) has a smaller diameter than the downstream end (closer to the operator). This safety-focused design ensures the device fits the sinus well and minimizes the risk of device embolization. Figure 2 depicts the device and its position within the heart.^25^

**Figure 2:** Characteristics and anatomical position of the coronary venous sinus reducer in the coronary sinus. The image on the left illustrates the anatomical position in the coronary venous sinus where the device will be implanted. On the right, there is an image of the expanded mesh in the shape of an hourglass inside the sinus with its balloon catheter.

Adapted from Verheye et al.

### Pathophysiological rationale

In a healthy, non-ischemic heart, blood flow is typically greater in the subendocardial myocardium than in the subepicardial myocardium. During periods of high demand, the sympathetic nervous system stimulates vasoconstriction of the subepicardial coronary arteries, which enhances blood perfusion in the subendocardial coronary bed and improves muscle contractility. However, when there are significant obstructions in the epicardial coronary arteries, blood flow is redistributed from the subendocardium, where there is greater resistance, to the subepicardium, which has less resistance.^26^ As a result, the perfusion of the subendocardial region is compromised, leading to ischemia. This can cause symptoms such as angina, reduced contractility, and ventricular dysfunction, subsequently increasing the left ventricular end-diastolic pressure (LVEDP).^27^ Elevated LVEDP exerts external pressure on the subendocardial capillaries and arterioles, further increasing their vascular resistance, which worsens the perfusion deficit and intensifies ischemia.^27,28^

When the coronary venous sinus is narrowed, it retrogradely increases pressure within the venous and capillary system. This results in slight arterial vasodilation, a key factor in the reduction of subendocardial vascular resistance. This, in turn, improves ischemia, contractile function, and related symptoms.^28-31^ Recent studies indicate that the CSR also has a direct impact on coronary microcirculation. In patients experiencing angina with unobstructed coronary arteries, the implantation of CSR has been demonstrated to enhance coronary flow reserve and decrease the index of microvascular resistance at the six-month mark.^32^ Several mechanisms may contribute to changes in myocardial perfusion and microvascular resistance following CSR implantation. These mechanisms include the recruitment of collateral circulation, increased capillary recruitment and diameter, improved coronary-cardiac coupling, and neovascularization. Current research is actively exploring these mechanisms.^33^ Figure 3 illustrates the mechanisms of subendocardial perfusion and ischemia before and after the narrowing of the venous sinus.

**Figure 3:** Pathophysiology of ischemia and contractile deficit before and after narrowing of the coronary venous sinus. A) In a non-ischemic heart, blood flow in the subendocardium is greater than in the subepicardium. During exercise, selective sympathetic-mediated vasoconstriction of the subepicardial vessels causes increased subendocardial perfusion and ventricular contractility. B) In an ischemic heart due to a significant stenosis of the epicardial coronary artery, myocardial perfusion is redistributed to the subepicardial layers, reducing perfusion to the subendocardium during stress. This reduced perfusion results in ischemia, contractile deficit, and elevated left ventricular end diastolic pressure (LVEDP). C) Narrowing the coronary venous sinus leads to increased retrograde pressure in the coronary venous system, which dilates the arterioles, reduces vascular resistance in the subendocardium, and improves blood flow. LV: left ventricle; CSR: coronary sinus reducer; CV: capillary venous.

Adapted from Gindi et al.^28^

### Technical aspects

The procedure is minimally invasive and performed within the venous system. It begins with an ultrasound guided venous puncture and sheath placement, typically via the right internal jugular vein. A catheter is then advanced over a guidewire into the coronary venous sinus. Mean right atrial pressure is measured, and if it is less than 15 mmHg, a coronary sinus venogram is performed to confirm suitable anatomy prior to device implantation.

The currently commercially approved device by CE Mark in European Union and United Kingdom is the Neovasc Reducer (Neovasc Inc.). This device contains three radiopaque anatomical markers: one proximal and one distal to the balloon, indicating its limits, and another more proximal marker, located just outside the balloon. The distal marker helps the interventional cardiologist assess the balloon's distal position, as illustrated in Figure 4.

**Figure 4:** Characteristics of the coronary sinus reducer device. The figure illustrates the guide catheter with its balloon and mesh expanded in an hourglass shape and the radiopaque anatomical markers.

Adapted from Dimitriadis et al.^31^

The device is mounted on a balloon and is available in a single size with variable expansion capabilities. It is inflated to 10% to 20% above the reference venous diameter, requiring approximately 4 to 6 ATM of pressure. This expansion causes the balloon's ends to anchor securely in the venous endothelium, with distal and proximal ends expanding to diameters of up to 9 mm and 14 mm, respectively. The central stenotic area has a diameter of approximately 3 mm, representing about 60% to 70% of its original size (7 to 13 mm). Notably, the device should ideally be positioned 2 to 4 cm distal to the coronary sinus ostium.

After implantation, a local inflammatory reaction begins, leading to endothelialization of the metal mesh at its ends, where it contacts the endothelium. This process typically occurs four to six weeks after the procedure and leads to a pressure gradient, resulting in improved ischemic symptoms,^4,31,34^ (Figure 5), which shows the angiographic results before and after the CSR implantation.

**Figure 5:** Coronary venous sinus angiographic image with the reducing device. The left image illustrates the coronary venous sinus before the coronary sinus reducer is implanted. The other images show the reducer's final hourglass appearance after implantation.

Taken from Konigstein et al.^37^

To minimize the risk of thrombotic events, patients are treated with dual antiplatelet therapy (DAPT) using acetylsalicylic acid (100 mg) and clopidogrel (75 mg) for six months after the procedure. However, it is important to note that no studies have yet compared CSR outcomes with and without DAPT.^4^

Potential complications from the procedure resemble those associated with central venous access, including bleeding, arterial puncture, cervical hematoma, and pneumothorax, which may be managed conservatively, with blood transfusion, or with chest drainage, respectively. Device-related complications are uncommon, but may include coronary venous sinus dissection, perforation, device migration, and complete occlusion.^35^ These issues can be promptly identified via fluoroscopy or further evaluated using computed tomography angiography. Coronary sinus dissection or perforation is generally manageable with conservative measures or minimally invasive procedures such as pericardiocentesis. Device migration may be asymptomatic or present with symptoms like chest pain and dyspnea, and is typically managed conservatively, via percutaneous retrieval, or – if these approaches fail – through surgical removal. The risk of device migration increases when the sinus diameter exceeds 13 mm or when the balloon expansion is insufficient to anchor the device to the endothelium. Although no cases of device thrombosis have been reported, the consensus among experts supports the use of DAPT.^4,31,35^

The A-Flux™ system, a novel device developed by VahatiCor, is currently undergoing clinical evaluation and is not yet commercially available. The A-Flux incorporates several technical advancements over balloon-expandable systems that may enhance both safety and efficacy. It is constructed from a dense nitinol mesh and is available in four different sizes, allowing for implantation in the specific segment of the coronary sinus where the greatest therapeutic benefit is anticipated. The dense nitinol structure is designed to promote rapid flow modulation through an accelerated endothelialization response. Additionally, the delivery system enables a low, chronic outward deployment force, securing the device in place without the risk of balloon-induced coronary sinus dissection or rupture.^36^

As of now, neither the A-Flux™ nor the Neovasc Reducer™ is available in Brazil. The Neovasc Reducer™ is currently undergoing an FDA-approved clinical trial in the United States.

### Scientific evidence

Numerous studies have demonstrated the safety and effectiveness of various interventions in reducing the intensity and frequency of angina in the short and long term. This evidence is derived from a combination of unblinded non-randomized and blinded randomized studies.

Initially, Banai et al.^34^ revisited Claude Beck's concept and conducted preclinical studies in pigs with stenosis of the proximal circumflex artery.^34,37^ These studies examined conditions with and without ischemia using stress echocardiography. The results demonstrated a reduction in the extent and severity of the ischemic area, as assessed by dobutamine echocardiography. Additionally, the studies confirmed the feasibility and safety of the procedure. Histological analysis conducted on the animals after one to six months showed an acceptable local inflammatory response, complete endothelialization of the device, and stable neointima.

The same research group published the first clinical study on catheter-based coronary sinus reduction in humans involving 15 patients.^34^ This was an open-label, non-randomized clinical trial to evaluate the safety and feasibility of percutaneous CSR implantation in patients with RA, classified as Canadian Cardiovascular Society (CCS) angina functional class 2 or higher. The trial reported no significant adverse events during the six-month follow-up period. Efficacy was measured using the CCS class, while ischemia was assessed through dobutamine stress echocardiography and thallium positron emission tomography (PET). The mean angina class decreased significantly from 3.07 to 1.64 (p < 0.001), and improvements in both the extent and severity of ischemia were noted (p = 0.004 and 0.042, respectively).

Konigstein et al.^38^ conducted a non-randomized study involving 23 patients and demonstrated improvements in angina, ischemia, and left ventricular contractility in patients with RA.^38^ Intervention was technically successful in 21 out of 23 patients. Statistically significant clinical benefit was documented by a reduction in the mean CCS score from 3.3 at baseline to 2.0 at six months, an increase in exercise duration from 3:16 to 5:16 min, and a reduction in the ^201^Tl-SPECT summed stress score and summed difference (21.5±10 vs.13.2±9, and 11.1±6 vs. 4.7±4, respectively), demonstrating the importance of the technique as novel therapeutic strategy for treating patients with RA.

The COSIRA trial,^25^ published in 2015, was the first randomized, placebo-controlled study that included 104 individuals with RA classified as CCS class 3 and 4, experiencing myocardial ischemia.^25^ The study found that the device led to an improvement of at least one angina functional class in 71% of patients in the intervention group (37 out of 52) compared to 42% in the placebo group (22 out of 52) (p = 0.003). Additionally, at least two angina classes improved in 35% of the intervention group (18 out of 52) versus 15% in the placebo group (8 out of 52) (p = 0.02). While it is important to note the significant rate of improvement in the placebo group, the intervention showed a 2.4 times greater reduction in angina class compared to the control, indicating its superiority as an antianginal treatment. A post-hoc analysis of the COSIRA trial cohort also revealed improvements in quality of life and exercise tolerance within the intervention group, further supporting the effectiveness of the intervention.^39^

In another single-center observational study, Giannini et al.^40^ evaluated 50 patients with RA and documented ischemia, focusing on angina, exercise tolerance, and quality of life at fuor4 and 12 months of follow-up.^40^ The procedure achieved a technical success rate of 100%, with no device-related adverse events reported. Among the patients, 80% experienced a reduction of at least one CCS class, and 40% experienced a reduction of at least two classes, with a mean class reduction to 1.67 ± 0.83 vs. 2.98 ± 0.52 (p < 0.001) at 4-month follow-up compared to baseline. There were also significant improvements in quality of life and exercise tolerance, as measured by the six-minute walk test, along with decreased need for antianginal medications. Importantly, these improvements were sustained over time, even after the first year of follow-up.

The same group further explored the symptomatic effects of CSR in patients with angina due to ANOCA in a prospective observational study involving eight patients classified as CCS angina classes 3 and 4.^41^ The procedure was successful in all cases, and no adverse events were reported. The CCS class improved from a median of 3.0 to 1.5 (P = 0.014). After one year of follow-up, three out of five patients examined continued to show benefit. Furthermore, improvements were observed in the 6-minute walk test and the angina symptoms, as measured by the Seattle Angina Questionnaire (SAQ).^41^

In a prospective registry study involving 48 patients with CCS 3 and 4 RA, Konigstein et al.^42^ evaluated improvements in angina using the SAQ, CCS functional class, and objective assessments of angina and ischemia. These assessments included treadmill stress tests, dobutamine echocardiography, and six-minute walk tests. The findings demonstrated that 85% of patients (33 out of 39) experienced an improvement of at least one CCS functional class, while 48% (19 out of 39) saw an improvement of at least two classes. Regarding ventricular function, the left ventricular ejection fraction (LVEF) under stress increased from 51±10 to 56±10 (p = 0.004), and the parietal motion index improved from 1.58±0.40 to 1.37±0.30 (p = 0.004), indicating positive effects on ventricular function. No procedure or device-related events were reported during a mean follow-up period of 12.5 months.

Subsequently, Tzanis et al.^43^ conducted a study focusing on symptoms and ventricular function in 18 patients, finding a reduction in one CCS class in 84% of subjects after four months of follow-up.^43^ There were also significant improvements in left ventricular function, with increases in LVEF and reductions in left ventricular end-systolic and diastolic volumes. Notably, half of the patients had reduced LVEF at baseline, showing an even greater increase in LVEF compared to those with preserved LVEF (an increase of 11.3 for reduced LVEF versus 3.8 for preserved LVEF; p = 0.029).

Further investigation into the severity of coronary obstruction was carried out by Zivelonghi et al.^44^ in a retrospective multicenter study involving 205 patients, of whom 103 (50.2%) had non-revascularized coronary total occlusion (CTO). This allowed for comparing the effects of CSR in patients with and without CTO-type lesions. Initially, the CCS class was 3.0±0.5 in the CTO group and 3.1±0.6 in the non-CTO group (p = 0.45). At the six-month follow-up, the CCS classes improved to 1.6±0.9 and 2.0±1.1, respectively (p < 0.01), with significantly greater improvement observed in the CTO group (1.4±0.9 vs. 1.1±1.0; p = 0.01). Angina improvement was observed in all territories including 70% for right coronary artery CTO (35 patients), 85% for left anterior descending artery CTO (17 patients), and 82% for circumflex artery CTO (27 patients). The conclusion was that patients with CTO lesions may respond better to CSR than non-CTO patients, regardless of the affected territory. No procedure-related complications were reported, although device embolization occurred in two cases (1%), where the device was successfully retrieved, and the procedure was completed with the implantation of a second CSR. Overall, similar adverse events were noted in both study groups, including a total of seven cardiac deaths (3.4%) and 30 additional revascularizations (14.6%) due to persistent angina.

The long-term benefits of CSR were also explored in the REDUCER-I study, a multicenter, observational study with both retrospective (from the COSIRA trial) and prospective arms.^45^ This study included 228 patients with CCS classes 2 to 4 RA of whom 180 were in the prospective arm and 48 in the retrospective arm, and it was aimed to assess the safety and efficacy of CSR and its role in improving angina severity and quality of life at a two-year follow-up. The success rate was found to be 99%. The average CCS class decreased from 2.8±0.6 to 1.8±0.7 after two years; at least one CCS class improved in 82% of patients, and at least two classes improved in 31% of patients. Significant increases were also observed in the six-minute walk test and the exercise duration. The non-responder rate was 24% at one year and 17% at two years. Improvements were seen in all domains measured six and twelve months after treatment. Notably, the study highlighted the sustained improvement in limiting angina from six months to two years of follow-up.

The RESOURCE study represents the largest multicenter registry, evaluating CSR implantation procedures over 10 years (from 2010 to 2020).^35^ It included data from 658 patients with RA, of whom 89.4% had a CCS class 3 or 4. The mean follow-up for these patients was 502 days. The study assessed various outcomes, including improvements in angina, the success rate of the procedures, procedural complications, and major cardiovascular events (MACE). The intervention was successful in 96.7% of cases and suspended in 3% of cases due to inadequate anatomy or complications. Still, there were no serious events in the first 48 hours. Per-procedure complications occurred in 5.7% of procedures (38 out of 663). Among these complications, the most common were device embolization, cervical hematoma, coronary sinus dissection, disconnection of the device from the guide catheter before implantation, and coronary sinus perforation. Importantly, none of these complications resulted in per-procedure deaths or myocardial infarctions and did not lead to an increase in mortality or MACE during follow-up. The primary efficacy endpoint, which was reducing at least two CCS angina classes, was achieved in 39.7% of patients, while at least one class improvement was noted in 76.0%. The non-responder rate was 24%. The frequency of MACE was 14.6%, and the mortality rates at 1, 3, and 5 years were 4.0%, 13.7%, and 23.4%, respectively. These figures are comparable to mortality rates observed in RA patients in the OPTIMIST cohort.^9^

In a recent meta-analysis of nine studies (n=846), including one randomized placebo-controlled trial (COSIRA), Hochstadt et al.^46^ demonstrated a combined efficacy of the CSR of 76% for improving symptoms in at least one CCS class of CAD and 40% for at least two classes. Additionally, the average distance walked during a six-minute walk test increased by 45.5 meters, exceeding the improvement in heart failure studies (30.1 meters). When it comes to safety, the procedure was successful in an impressive 98% of cases. Only 3% of patients experienced complications, such as sinus perforation/dissection, embolization or displacement of the device, and periprocedural infarction. Importantly, none of these complications resulted in cardiac tamponade or death, further confirming the procedure's high success rate and safety.

In 2024, the highly anticipated findings of the ORBITA-COSMIC trial were published.^47^ This landmark randomized, double-blind, placebo-controlled clinical trial involved 51 patients and aimed to evaluate the impact of CSR on myocardial ischemia. The analysis involved quantifying myocardial blood flow via cardiac magnetic resonance (CMR) imaging under adenosine stress, conducting exercise testing, and utilizing a real-time symptom questionnaire through the study smartphone application ORBITA-app. The primary outcomes included myocardial blood flow in ischemic segments, as determined by CMR, and the primary symptom outcome was the daily number of angina episodes. The trial did not demonstrate an improvement in myocardial blood flow to ischemic segments six months after CSR implantation. However, there was a proportional improvement in endocardial compared to epicardial blood flow under stress in the CSR group and there was a difference in this effect between ischemic and non-ischemic segments. Patients in the CSR group reported fewer daily episodes of angina at the six-month follow-up beginning at around 70 days post-procedure, aligning with the timeline for device endothelialization and the formation of a pressure gradient starting six weeks after CSR implantation. For the secondary outcomes, there was an improvement in angina frequency based on the SAQ, although no statistical differences were found in quality of life and exercise tolerance. In this study, the symptoms’ improvement in patients with RA was not accompanied by an overall improvement in perfusion of ischemic segments. On the other hand, there was an improvement in subendocardial perfusion within the ischemic segments, supporting the theory that the device redistributes blood flow towards the endocardium.^48^ These findings have important implications for the understanding of myocardial ischemia and the potential role of CSR in its treatment.

### Patient selection

The scientific evidence supporting CSR indicates it is suitable for treating angina pectoris or ischemic equivalents in patients with chronic obstructive coronary syndromes. This treatment is specifically indicated for patients with RA who have not responded to optimal medical therapy, which includes at least two or three classes of anti-anginal medications, and who have no or limited surgical or percutaneous revascularization options.^4^

Despite the benefits observed in various clinical studies, 20 to 30% of patients may not respond positively to this treatment.^49^ Various factors can contribute to an unfavourable clinical response,^50,51^ including non-anginal chest pain, angina due to ischemia in the right coronary artery territory, dyspnea on exertion resulting from heart failure, and inadequate coverage of the metal structure of the device, which can lead to poor endothelialization of the device struts.

Additionally, well-developed alternative myocardial venous drainage through a Thebesian network may affect outcomes.^52^ A biventricular pacemaker for cardiac resynchronization therapy may complicate the procedure from a technical standpoint, as its leads are positioned close to the sinus venosus ostium, increasing the risk of acute complications and potential device insertion failure. Moreover, advanced or symptomatic heart failure with elevated right atrial pressure (greater than 15 mmHg) may signal cardiac decompensation, further reducing the intervention's effectiveness. Table 1 outlines the positive and negative factors when selecting the ideal patient who may benefit from CSR treatment.

**Table 1:** Criteria for selecting ideal candidates for coronary sinus reducer therapy

Suitable Candidates	Unsuitable Candidates
Stable angina (> 3 months), CCS class ≥ 2 despite optimal medical therapy	Non-coronary chest pain (severe valvular heart disease, heart failure, non-cardiac pain)
Evidence of reversible ischemia	Absence of stress-induced ischemia
Myocardial ischemia at the left coronary artery territory	Myocardial ischemia exclusively at the right coronary artery territory
Left ventricular ejection fraction > 30%	Left ventricular ejection fraction < 30% or heart failure NYHA class ≥ 3
Mean right atrial pressure or central venous pressure < 15 mmHg	Mean right atrial pressure or central venous pressure > 15 mmHg
Pacemaker leads implanted in the right atrium > 3 months	Pacemaker leads implanted in the right atrium < 3 months
	Cardiac resynchronization therapy leads in the coronary sinus
	Recent acute coronary syndrome, percutaneous coronary intervention or bypass surgery < 3 months

Adaptado de Konigstein et al.^52^ CCS: *Canadian Cardiovascular Society*, NYHA: *New York Heart Association*.

### Perspectives

Previous observational studies suggest that the CSR may provide clinical benefits for patients with ANOCA and coronary microvascular dysfunction. These benefits include symptom relief and improvement of ventricular function in heart failure patients with preserved and reduced LVEF. This opens a fascinating array of potential new therapeutic options for patients suffering from non-obstructive chronic coronary syndromes and non-coronary cardiomyopathies, such as hypertrophic cardiomyopathy.

Several clinical trials listed in Table 2 are currently underway, each playing a crucial role in advancing our understanding and the benefits of the CSR in different groups of patients with RA.

**Table 2:** Current studies involving the coronary sinus reducer

Study Name	Country	Type of Study	Population	Primary Outcomes	NCT
LSSRR – Lower Silesia Sinus Reducer Registry^53^	Poland	Observational	Patients with chronic disabling refractory angina pectoris (CCS classes 2-4) despite maximally tolerated anti-angina medical therapy who underwent CSR implantation	CCS class, SAQ-7 scores, 6-MWT, and echocardiography at 1 year after the implantation of the CSR	NCT06288165
Impact of Coronary Sinus Flow Reducer on Coronary Microcirculation and Myocardial Ischemia^54^	Croatia	Interventional, single group	Patients with coronary artery disease and refractory angina pectoris who are ineligible for coronary revascularization	Assessment of coronary flow reserve and index of microcirculatory resistance before and after CSR implantation	NCT06266065
Use of the Neovasc Coronary Sinus Reducer System for the Treatment of Refractory Angina Pectoris in Patients With Angina Class 3-4 Who Are Not Candidates for Revascularization (Reducer)^55^	Israel	Interventional, single group	Symptomatic CAD with chronic refractory angina pectoris classified as CCS grade III or IV despite attempted optimal medical therapy for thirty days prior to screening Non-candidate for surgical or PCI Reversible ischemia of the center ventricular wall demonstrated by dobutamine stress echocardiography or by Thallium SPECT	A decrease of two or more CCS grades from baseline to six-month post-procedural	NCT01566175
COSIMA: COronary SInus Reducer for the Treatment of Refractory Microvascular Angina (COSIMA)^56^	Germany	Randomized, controlled, parallel-groups (CSR vs. OMT)	Chronic coronary syndrome (including patients with anginal equivalents) refractory angina CCS class III-IV despite guideline-directed medical therapy Evidence of reversible ischemia on non-invasive testing Evidence of microvascular disease as diagnosed invasively by at least one of the following: IMR >25 and/or CFR <2.0 with FFR >0.8	Proportion of patients with improvement of ≥ 2 classes of angina by CCS six months after RSC implementation	NCT04606459
IMR Evaluation in Patients With Coronary Sinus Reducer Implantation (INROAD Study)^57^	Italy	Interventional, single group	Chronic refractory angina to medical and interventional therapies At least one open coronary artery (excluded right coronary artery) where to performs IMR evaluation	≥ 20% change in IMR value at 4 months after CSR implantation compared to baseline	NCT05174572
Efficacy of the COronary SInus Reducer in Patients With Refractory Angina II (COSIRA-II)^58^	United States	Interventional, randomized, double-blinded, sham-controlled	Symptomatic CAD with persistent refractory angina pectoris classified as CCS Grade III or IV despite maximally tolerated OMT Evidence of either exercise or pharmacologically induced reversible ischemia severity by stress echo, nuclear study, PET, perfusion MRI, CT perfusion, FFRCT, FFR, iFR, or other non-hyperemic tests in the distribution of the center coronary artery	Change in total exercise duration in a modified Bruce treadmill exercise tolerance testing evaluation in the treatment arm compared to sham-control arm The rate of occurrence of a composite of death, MI, pericardial effusion requiring surgical or percutaneous intervention, device embolization, or BARC 3 or 5 bleeding evaluation in the treatment arm compared to the sham-control arm	NCT05102019
Coronary Sinus Reducer Implantation in Patients With Ischaemia and Non-obstructed Coronary Arteries and Coronary Microvascular Dysfunction (REMEDY-PILOT)^59^	Reino Unido	Intervencionista, randomizado, duplo-cego, controlado por procedimento simulado (sham)	Ongoing symptomatic angina, CCS Class II-IV, for ≥3 months despite background treatment with at least two anti-anginal drug at the maximal tolerated dose; Unobstructed coronary arteries with ≤30% epicardial stenoses demonstrated on coronary angiography; Circumferential subendocardial stress-induced hypoperfusion on CMR (global MPR < 2.5)	Change at 6 months post randomisation, compared to baseline, in quantitative global MPR assessed by cardiac MRI	NCT05492110
Myocardial Ischemia After Coronary Sinus Reduction Stent Implantation (MICS-Reduce)^60^	Norway	Observational	CAD and refractory angina	Change in myocardial flow reserve on ^15^O-H_2_O PET/CT	NCT06033495

CSR: coronary sinus reducer, CCS: Canadian Cardiovascular Society, NYHA: New York Heart Association, SAQ-7: Seattle Angina Questionnaire - 7 items, 6-MWT: 6-minute walk distance test, CAD: coronary artery disease, PCI: percutaneous coronary intervention, SPECT: Single-photon emission computed tomography, OMT: optimal medical therapy, IMR: index of microvascular resistance, CFR: coronary flow reserve, FFR: fractional flow reserve, echo: echocardiography, PET: positron emission tomography, MRI: magnetic resonance imaging, CT: computed tomography, FFRCT: fractional flow reserve derived by computed tomography, iFR: flow reserve index, CMR: coronary microvascular resistance, MPR: myocardial perfusion reserve, MI: myocardial infarction.

Author source.

## Conclusion

The CSR is a device inserted percutaneously through a minimally invasive procedure. It is both safe and effective for controlling ischemic symptoms in patients with RA who are not candidates for surgical or percutaneous revascularization and are receiving the best medical therapy. The device, with its proven safety and effectiveness, is thought to work by retrogradely increasing pressure in the coronary venous system, which helps improve arteriocapillary perfusion in the subendocardial myocardium.

Blinded data indicate angina improvement with CSR implantation. It also enhances the quality of life for patients with no other options. However, it is crucial to note that 20 to 30% of patients may not respond to the treatment. Therefore, the procedure should be carefully indicated based on thorough clinical and anatomical assessments. Furthermore, small unblinded studies suggest that CSR may improve perfusion and angina symptoms in patients with ANOCA and coronary microvascular dysfunction and enhance myocardial contractility in heart failure cases. However, the need for further randomized, placebo-controlled studies is vital to evaluate and validate the efficacy of CSR more thoroughly and to advance our understanding of this promising treatment.

Finaly, after 70 years since the first coronary sinus reduction performed the Beck´s surgery, scientists have developed a minimally invasive procedure that has been successfully tested in randomized controlled trials for the treatment of angina. This procedure has recently been included in clinical guidelines. The Central Illustration represents this evolution, highlighting key milestones.

## Data Availability

The content is available at the: doi:10.1016/j.jtcvs2014.06.032 doi:10.1056/NEJMoa1402556 doi:10.1016/j.carrev.2022.01.003 doi:10.1016/j.carrev.2023.07.008 doi:10.1093/eurhearttj/ehx486
